# A Ceramic Network for Hybrid Solid Electrolyte Lithium Metal Batteries

**DOI:** 10.1002/advs.76063

**Published:** 2026-07-02

**Authors:** Luca Weckelmann, Jeong Seop Yoon, Jehad Ahmed, Krzysztof Dzieciol, Anna Windmüller, Luc Raijmakers, Sanja Tepavcevic, Hans Kungl, Venkat Srinivasan, Chih‐Long Tsai, Rüdiger‐A. Eichel

**Affiliations:** ^1^ Institute of Energy Technologies Fundamental Electrochemistry (IET‐1) Forschungszentrum Jülich Jülich Germany; ^2^ Institute of Physical Chemistry RWTH Aachen University Aachen Germany; ^3^ Chemical Sciences and Engineering Division Argonne National Laboratory Lemont Illinois USA; ^4^ Materials Science Division Argonne National Laboratory Lemont Illinois USA; ^5^ Argonne Collaborative Center For Energy Storage Science Argonne National Laboratory Lemont Illinois USA

**Keywords:** all‐solid‐state battery, ceramic network, hybrid solid electrolyte, lithium metal anode, tortuosity

## Abstract

Hybrid solid electrolytes (HSEs) represent a promising material system for lithium metal batteries by combining high ionic conductive and mechanically strong inorganic with soft organic solid electrolytes. However, for HSEs consisting of ceramic fillers, lithium hopping between the polymer and ceramic phases remains unproven, emphasizing the tortuosity inside the polymer phase as a pivotal factor. Herein, we report an in‐plane aligned Li_6.6_La_3_Zr_1.6_Ta_0.4_O_12_ (Ta‐LLZO) ceramic fiber network structure for HSEs and compare its morphological nature to typical LLZO‐based fillers. Simulations reveal a high tortuosity inside the polymer phase with a parallel to the electrodes aligned network structure, enabling dendritic structure blocking behavior, but still reaching decent ionic transport (0.44 mS cm^−1^, 60°C). Post‐mortem microscopy analyses confirmed the dendrite blocking mechanism of the network, ultimately leading to long cycling lifetime of > 1100 h in symmetric lithium metal cells operated with 0.1 mA cm^−2^ at 60°C. Overall, the ceramic network HSE represents a superior morphology by combining decent ionic conductivity, low resistance and long cycling life in symmetric and full cells with LiFePO_4_ cathodes. The comparison of different filler characteristics with a special focus on tortuosity eventually facilitates the evaluation of these properties for the purpose of optimizing HSEs.

## Graphical abstract

An in‐plane aligned Ta‐LLZO ceramic network exhibits superior performance compared to conventional fillers, including single ceramic fibers and particles. The tortuosity within the polymeric phase of hybrid solid electrolytes is identified as a key parameter governing lithium‐ion transport pathways and dendrite suppression.

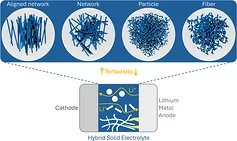

## 1 Introduction

Batteries are one of the key technologies for advancing global electrification. While conventional lithium‐ion batteries (LIBs) are reaching their theoretical energy densities and show safety concerns, next generation batteries such as all‐solid‐state lithium metal batteries (SSLBs) can overcome the energy ceilings and safety issues [1]. Due to the use of a lithium metal anode, a drastically increased theoretical gravimetric (1088 Wh kg^−1^) and volumetric (5211 Wh L^−1^) energy density compared to the commercially used intercalation graphite anodes (696 Wh kg^−1^, 1928 Wh L^−1^) paired with cathodes, such as lithium nickel manganese cobalt oxides (NMC), can be enabled [2]. Although SSLBs are, therefore, often regarded as one possible successor of LIBs, superior SSLBs are not commercially available yet. Dendrite formation is one of the main causes that hinders the operation of SSLBs at sufficiently high current densities [3, 4, 5]. Mechanical imperfections, such as uneven surfaces or flaws, chemical side reactions due to highly reactive lithium or electrochemical induced decomposition can lead to inhomogeneous lithium plating and stripping at the anode, eventually leading to parasitic lithium metal growth. Besides that, the low lithium self‐diffusion cannot catch up with high discharging rate, ultimately creating voids and point contacts during stripping, causing dendrite formation at later time during charging [6]. To minimize and prevent the underlying mechanisms for dendrite growth, various solid electrolytes in combinations with lithium metal anodes are being researched. Yet, different materials involve different advantages and disadvantages. While oxides e.g. Li_7_La_3_Zr_2_O_12_ (LLZO) exhibit high ionic conductivities and a wide electrochemical window, their rigid ceramic nature leads to poor interfacial contact with lithium [7]. Sulfides are softer compared to oxides and can achieve even higher ionic conductivities but suffer from reaction with lithium metal [8, 9]. An even better interfacial contact can be accomplished with solid‐state polymers. However, traditional solid polymers often come with low ionic conductivities at room temperature, limiting their performance significantly while dynamic polymers can provide higher conductivities [10, 11].

For that reason, the combination of two different solid electrolyte types presents a highly promising approach [12, 13]. Such systems are based on the idea of using the individual advantages and equalizing the disadvantages at the same time. Hybrid solid electrolytes (HSEs) usually consist of a polymer matrix with an inorganic filler part. The polymer part ensures good interfacial contact with the electrodes and the inorganic part may provide a high ionic conductivity path and strengthen the mechanical properties of the hybrid system. For both to take place, a polymer layer needs to be in contact with both solid electrolyte/electrode interfaces and lithium ions need to move within the inorganic and organic parts. The former is highly dependent on the filler fraction and size of the inorganic part. For example, it was found that a larger filler size can lead to rougher surfaces, which complicates to establish a flawless interfacial contact [14]. Whether long range ionic conduction occurs inside both organic and inorganic parts depends on the interfacial resistivity for hopping between the two phases. However, even if hopping between the two phases is energetically not favorable, filler can improve the ionic conductivity by suppressing the commonly lower ionic conductive crystalline phase of the polymer matrix, improving salt dissociation or increasing the ion mobility at the inorganic/organic interface [15, 16, 17, 18]. For all three possible ionic pathways (polymer phase, inorganic phase, both phases), the filler fraction, size, morphology, and orientation influence the overall conduction behavior. All these properties of the inorganic filler determine the tortuosity of the lithium ion pathways and thus the effective ionic pathway through the solid electrolyte. In case of an arising filler effect, the surface‐to‐volume ratio (SVR) additionally advances to a decisive parameter and determines the size of the interfacial area. Like the crucial impact on the ionic conductivity, the inorganic filler properties have also a crucial influence on the mechanical features and dendrite tortuosity/deflecting behavior of the hybrid system [19]. It was reported that ceramic particles with a 200 nm diameter exhibit a higher conductivity than 5 µm‐sized particles, but the latter led to longer cycle life in symmetric cells [20]. For both diameters, a higher filler fraction increases the cycle life of the batteries but also significantly increases the polarization as well as polarization fluctuations. Overall, tortuosity represents a pivotal parameter, influencing the ionic conductivity as well as dendrite propagation.

Typical inorganic fillers for HSEs are oxide‐based ones, such as Al‐ or Ta‐substituted lithium lanthanum zirconate oxide (LLZO). The used filler morphologies are commonly either particles [20, 21, 22, 23, 24, 25, 26, 27, 28, 29, 30, 31], fibers [32, 33, 34, 35, 36] or both [37, 38, 39, 40, 41, 42]. Besides those filler shapes, HSEs with interconnected LLZO fibers [43, 44, 45] or garnet ceramic skeletons [46] are reported. The ionic conductivities of LLZO‐based hybrid systems are typically in the order of 10^−4^–10^−3^ S cm^−1^ at room or elevated temperatures (40°C–80°C) for both particles and fibers as the fillers. A comparison between undoped, Al‐ and Ta‐substituted LLZO fibers revealed the highest conductivity for 5 wt.% Ta‐substituted LLZO fiber in a polyacrylonitrile (PAN) LiClO_4_ polymer matrix [39]. Similarly, it was reported that 10 wt.% fiber (LLZO) or particles (Ga‐LLZO) exhibit higher ionic conductivities compared to 20 wt.% filler in polyethylene oxide (PEO) with lithium bis(fluorosulfonyl)imide (LiTFSI) [38]. A further increase of the filler fraction from 20 wt.% leads to a decrease in ionic conductivity as it was shown by Zhang et al. [26] with Ta‐LLZO particles in PEO+LiTFSI and by Li et al. [40] with LLZO fibers with liquid electrolyte soaked poly(vinylidenfluorid‐co‐hexafluorpropylen) (PVDF‐HFP)+LiTFSI. Those findings support the assumption that no significant long‐range diffusion through both ceramic and polymer phases takes place in the reported systems. Thus, higher fillers fractions increase the tortuosity of the lithium‐ion diffusion pathways within the HSE. Additionally, a higher filler fraction can hinder segmental motion of the polymer chains, which leads to lower ionic conductivity. On the other hand, however, a higher filler fraction can increase the mechanical stiffness of HSEs and fillers can act as physical barriers against dendrite formation [20, 24, 36]. Here, a significant increase in Young's modulus was measured by AFM for PEO incorporating LLZO nanofibers (1–10 GPa) compared to pure PEO (≤10 MPa) [36]. In fiber network‐based HSEs, randomly oriented fibers show lower tensile strength compared to in‐plane aligned fiber networks, in both the machine and transverse directions [47], leading to superior dendrite resistance for in‐plane aligned structures.

In this work, we present an in‐plane aligned Ta‐LLZO ceramic network‐based hybrid solid‐electrolyte for lithium metal batteries. A composite fiber network was obtained via electrospinning from a Ta‐LLZO sol–gel mixed with polyvinylpyrrolidone (PVP) and dimethylformamide (DMF) to form a solution. During a subsequent calcination process, the fiber network structure was synthesized. Vacuum infiltration of solved PEO+LiTFSI into the network structure followed by vacuum drying led to the final HSE membranes. The synthesized Ta‐LLZO network was structurally and microscopically characterized as well as the obtained HSEs. To exclude the influence of the used polymer and focus on the filler morphology and its impact, Ta‐LLZO particle‐ and fiber‐based HSEs were fabricated for comparison. In this study, particular emphasis is placed on the effect of tortuosity for lithium ions and dendrite propagation within the polymeric phase. The filler morphology impact was theoretically evaluated with tortuosity simulations and practically examined via electrochemical experiments. All solid electrolytes were electrochemically tested in symmetric lithium metal and full cells paired with lithium iron phosphate (LFP) as the cathode. Post‐mortem analysis of the ceramic network HSEs cells reveals the dendrite blocking property of such systems. An improved overall electrochemical performance of the ceramic network structure compared to particles and single fibers with the same polymer matrix is demonstrated. The connected fibers build a strong mechanical unit and exhibit a large surface area for enhancing the ionic conductivity, ultimately leading to a superior morphology for hybrid solid electrolytes. Finally, the theoretical and experimental comparison of different filler morphologies, sizes, and fractions underscores their significance and serves as a framework for designing and choosing the optimal filler.

## 2 Results and Discussion

### 2.1 Ceramic Fiber Synthesis and Characterization

**FIGURE 1:**
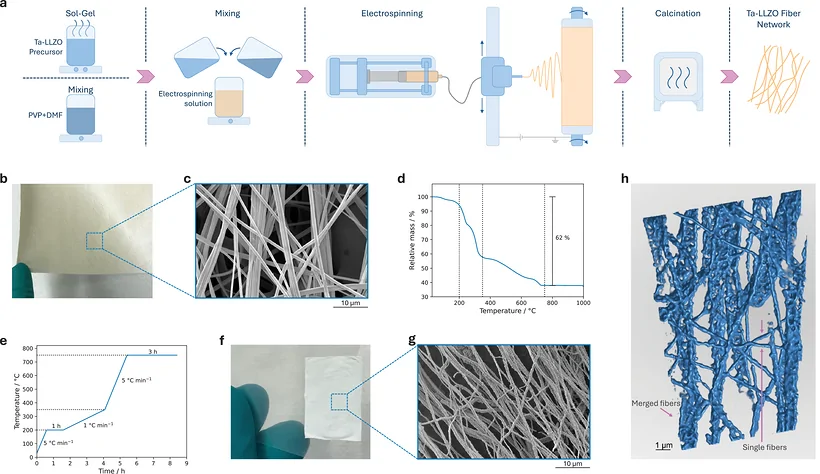
Ta‐LLZO ceramic network synthesis route. (a) Schematic illustration of the synthesis route. Optical image (b), SEM image (c) and TGA measurement (d) of a composite in‐plane aligned network mat. The dotted lines correspond to the temperatures in Figure 1e. (e) Heating protocol for the calcination process of the composite network mat based on the TGA and MS (Figure S1) measurement. Optical image (f) and SEM image (g) of a ceramic network plate. (h) Three‐dimensional renderings of the segmented ceramic network obtained from nXCT measurement.

A schematic illustration in Figure 1a depicts the fiber network synthesis route. Figure 1b shows an optical image of the composite fiber mat obtained via electrospinning. The “as spun” fiber mat consists of a carrier polymer mixed with the Ta‐LLZO precursor. Since electrospinning is conducted with a rotating drum as the fiber collector, the individual fiber alignment (Figure 1c) was achieved by changing the rotation speed of the drum to an optimized speed of 200 rpm. Here, the fibers exhibit a preferred orientation within the fiber mat plane. Thermogravimetric analysis (TGA, Figure 1d) of the as‐spun fibers was performed to reveal the critical temperatures, where significant mass loss happen and thus the ramping rate should be slow for avoiding combustion or rapid gas release to destroy the fiber structure. The major mass loss between 200°C and 350°C is due to the evaporation of the carrier polymer, which is indicated by mass spectroscopy (MS, Figure S1). Based on the TGA measurement, a calcination protocol with a maximum temperature of 750°C is developed (Figure 1e). Due to the starting of significant mass loss above 200°C, it is held for 1 h at 200°C and followed by a moderate heating rate of 1°C min^−1^ to avoid rapid evaporation of the polymer which could lead to cracks within the microstructure or the complete loss of the fiber structure. Figure 1f presents an optical image of a calcinated in‐plane aligned Ta‐LLZO fiber mat. As shown in the SEM image in Figure 1g, the fibers are partially merged and interconnected building a ceramic fiber network. The diameter of a individual ceramic fiber is < 1 µm but merged fiber structures can exhibit diameters around 1 µm and a highly porous structure. To further investigate the three‐dimensional morphology of the individual fiber in the nanometer range, a nano x‐ray computed tomography (nXCT) measurement was conducted (Figure 1h). Here, the merged fibers possess highly porous structures which enable pathways to be orthogonal to the fiber alignment direction (through holes). The Brunauer‐Emmett‐Teller (BET) method was used to determine the surface area of the fiber, which is crucial for lithium‐ion transportation along the interface. The resulting value of 1.02 m^2^ g^−1^ reveals a high surface area due to the porous nature of the structure. Assuming a density of 5.3 g cm^−3^ [48] and one cylinder with a diameter of 1 µm leads to a surface area of 0.75 m^2^ g^−1^. The measured surface area is comparable with a cylinder with a diameter of 750 nm (≈1 m^2^g^−1^).

**FIGURE 2:**
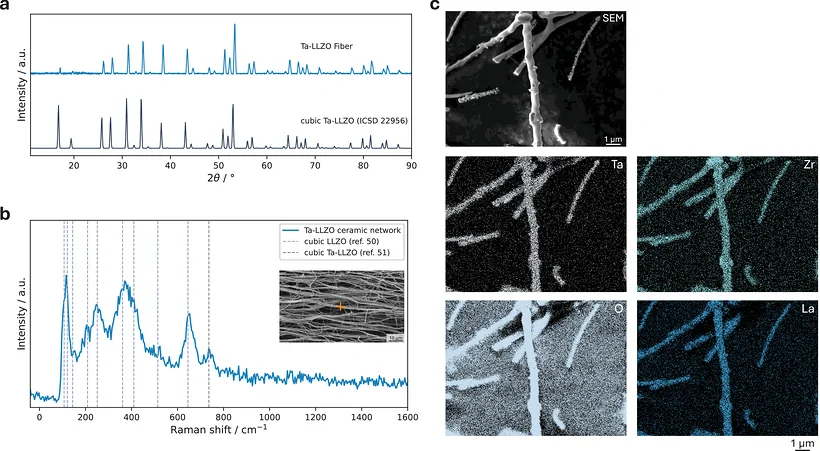
Structural characterization of the Ta‐LLZO ceramic network. (a) XRD diffractogram showing the pure cubic phase. (b) Raman spectrum showing the cubic Ta‐LLZO phase. (c) SEM image of single fibers that were EDS performed on and EDS maps for the elements tantalum (Ta), zirconium (Zr), oxygen (O) and lanthanum (La).

The x‐ray diffraction (XRD) pattern depicted in Figure 2a confirms that the ceramic fibers crystallized to the desired high ionically conductive cubic phase garnet structure. A temperature of 750°C with 3 h dwell time was determined to lead to the desired structure with the *Ia3d* space group. All peaks of the XRD pattern can be assigned to the peaks of Li_6.6_La_3_Zr_1.6_Ta_0.4_O_12_ (ICSD 22956) in the cubic phase [49]. Raman spectroscopy measurement (Figure 2b) shows that the ceramic network aligns with the spectrum of cubic LLZO [50], where the additional band at 736 cm^−1^ of TaO_6_ octahedra confirms the Ta substitution into LLZO structure [51]. Although the fibers were exposed to air for 1 h during the measurement, no LiCO_3_ was detected, indicating an air‐stability of Ta‐substituted LLZO. Figure 2c depicts an SEM image of one exemplary fiber with its elemental distribution (energy peaks Figure S2), measured with energy dispersive x‐ray spectroscopy (EDS). All elements are homogenously distributed throughout the fiber, indicating a successful Ta‐substituted LLZO synthesis through the sol–gel mixing of the precursor solution and the subsequent electrospinning process.

### 2.2 Hybrid Solid Electrolyte Synthesis and Characterization

**FIGURE 3:**
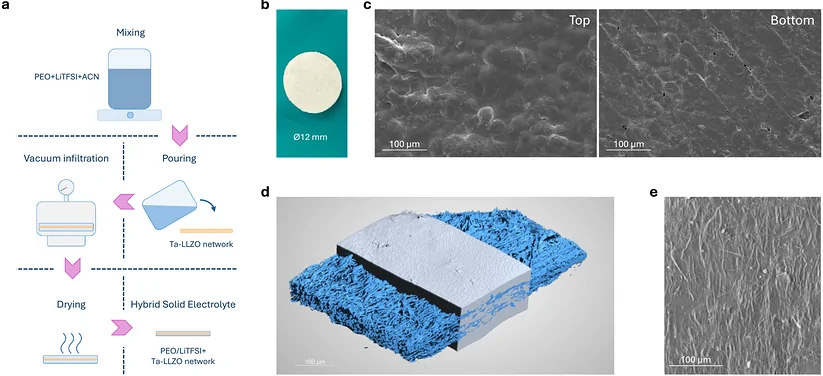
Microscopical characterization of the ceramic network HSE. (a) Schematic illustration of the HSE synthesis route. (b) Photo of a ceramic network HSE chip for use in a *Swagelok*‐type cell with a diameter of 12 mm. (c) SEM image of the top and bottom side. The top side is referring to the solid electrolyte side, which is pointing upwards and bottom to which is facing the PTFE dish side during drying. (d) Three‐dimensional rendering of the segmented µXCT scan. The ceramic network (blue) is fully embedded in the polymer matrix (grey). (e) Two‐dimensional in‐plane cross section recorded with µXCT.

Figure 3a depicts a schematic of the network HSE synthesis route. Here, the key step is the vacuum infiltration that enables complete infiltration of the PEO+LiTFSI solution into the network structure. Without vacuum infiltration, a polymer layer forms on top of and underneath the network structure, while only limited infiltration into the interstitial space is achieved, even at low solution viscosity. . One reason for this is the highly volatile solvent (acetonitrile) which is causing the solution to become viscous rapidly as well as the trapped gas within the fiber. Figure 3b shows a photo of a ø12 mm network HSE chip. As shown in the SEM images (Figure 3c), a solid polymer layer completely covers the top and bottom of the solid electrolyte membrane. Micro x‐ray computed tomography (µXCT) measurement verifies the successful infiltration of the polymer inside the network structure (Figure 3d, gray scale image Figure S3). No polymer‐free volume is visible between the fibers as shown in an exemplary in‐plane cross section, Figure 3e. Furthermore, the µXCT scan reveals an uncompressed, total network HSE thickness in the range of 130 µm, with an approximate thickness of 30 µm for the solid polymer layer situated beneath and above the 70 µm‐thick network structure. The synthesis route for hybrid solid electrolytes with single particles (µm‐range) and fibers is described in the experimental section. An overview of the HSEs synthesized in this work are listed in Table S1.

## 3 Simulation

**FIGURE 4:**
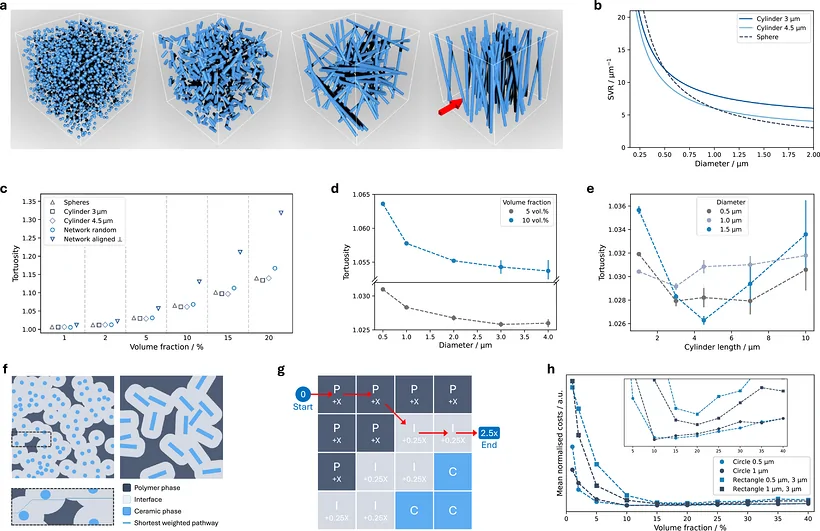
Tortuosity simulations for different fillers, sizes, and orientations. (a) Renderings of the simulated volumes with 5 vol.% spheres, cylinders, randomly oriented, and aligned network as filler. The red arrow indicates the simulation direction for the aligned network. (b) SVRs as a function of the diameter for spheres and cylinders with different lengths. (c) Simulated tortuosities of different filler types, randomly distributed for different volume fractions. (d) Simulated tortuosities of spheres as a function of their diameter for different volume fractions. (e) Simulated tortuosities of cylinders for different length‐diameter combinations. (f) Illustration of a two‐dimensional pathway through an HSE with three different phases with circles and rectangles as filler (10 vol.%). The allowed paths are only through the polymer phase and interface. (g) Visualization of the two‐dimensional cost simulation on pixel scale. The color labeling corresponds to the one of figure 4f while the red arrows indicate the ion hopping pathway, adding cost (energy) for every hop in the individual fields. (h) Results from the cost simulation for different volume fractions of circles and rectangles with different sizes.

To evaluate the impact of the filler fraction, size, morphology, and orientation on the tortuosity (see reference [52] for details on determining the tortuosity factor from voxelized microstructural data), simulations were conducted. The considered morphologies are particles (modeled as spheres), fiber (modeled as separated short cylinders), randomly oriented network (modeled as connected long cylinders), and aligned network (modeled as aligned cylinders) (Figure 4a from left to right). The dimensions are expressed in arbitrary units but were chosen to be of comparable magnitude to the underlying synthesized filler for better readability.

According to the assumption of a polymeric phase as the only conductive one and an increased ionic conductivity at the interfaces, the tortuosity inside the polymer phase and the surface area of the filler are crucial parameters. While a high filler volume can decrease the ionic conductivity through higher tortuosity, a higher surface area can increase the ionic conductivity. A connection between both can be established with the SVR. A high SVR is desired, which increases with decreasing diameter, for both cylinders and spheres (Figure 4b). Depending on the diameter of sphere and cylinder and on the length of the cylinder, either spheres or cylinders exhibit a larger SVR. Shorter cylinders come with higher SVR compared to longer cylinders and show larger SVRs until a diameter of 0.5 µm compared to spheres. Consequently, network structures, which consist of interconnected cylinders and thus significantly larger length, have lower SVRs compared to single fibers and particles due to the missing base area. A comparison of the tortuosities of different volume fractions for all three considered morphologies is shown in Figure 4c. Beside the expected increase in tortuosity with higher volume fraction for all morphologies, it gets obvious that the aligned network structure leads to the highest tortuosity for all volume fractions (movement direction perpendicular to fiber alignment). While the difference in tortuosity for 2 vol.% filler is not significant, it gets more pronounced for higher volume fractions. The high tortuosity values of the aligned network underline its capability of potentially blocking dendrites compared to other morphologies.

Different to the influence on the SVR, a smaller diameter of spheres results in higher tortuosity values, as illustrated in Figure 4d for different volume fractions. Interestingly, for cylinders, the tortuosity shows a minimum at certain length‐diameter‐combinations (Figure 4e). For example, the tortuosity is lower for a length of 4.5 µm and a diameter of 1.5 µm compared to the other investigated 0.5 and 1.0 µm diameters with different length of cylinders for a filler fraction of 5 vol.%. Nevertheless, it should be noted that bigger structures result in larger errors in the simulation, thus larger deviation. This can be caused by the increased dependence on the location of the individual filler. A larger structure concentrates more volume at one point and can thus have a larger impact on the tortuosity compared to smaller structures that can spatially distribute the same volume. Thus, the “good” or “bad” placement of large structures can significantly alter the tortuosity. This in turn can cause deviations of ionic conductivity for the same filler amount but also different surface roughness if certain particles are located close to the surface or not. Therefore, the performance of HSEs with large filler sizes is dependent on the distribution for the same filler fraction. In addition, this effect takes place if agglomerations of fillers appear. Especially fillers in the nanometer range are prone to agglomerate, resulting in a reduced total interfacial volume (lower SVR) [53]. Since a fiber network is rigidly connected, the individual fibers cannot agglomerate, which is a decisive advantage compared to single fillers (spheres, cylinders) that are prone to cluster. Moreover, the tortuosity is highly dependent on the fiber orientation. Spheres are rotational invariant, but a fiber orientation perpendicular to the direction of movement can significantly increase the tortuosity, as is the case for the aligned network structure.

The impact of the increased tortuosity and enhanced ionic conductivity at the inorganic/organic interfaces through the filler is discussed via a simplistic, qualitative two‐dimensional pathway simulation, as visualized in Figure 4f. The hopping costs (reflect the activation energy for hopping) are calculated for every individual pathway starting from every point on one side with the goal to reach the opposite side with minimal cost (Figure 4g). An algorithm (Dijkstra's algorithm) calculates the cheapest pathway (minimizing cost/energy). Figure 4h illustrates the mean costs for different filler fractions and different filler morphologies. Here, it needs to be considered, that the interface thickness and difference between the hopping cost inside the polymeric phase and at the interface alter the results for the same morphology and size. Simulations (Figure S4) with different interface thicknesses and ionic conductivity differences with various filler fractions lead to the following conclusions: A larger difference in ionic conductivity between the crystalline polymeric phase and the interface shifts the peak conductivity to lower volume fractions of fillers. The same applies to larger interfaces, which lead to a shift to lower volume fractions of fillers. The interface thickness is reported to be in the order of 2–3 times of the filler diameter [28]. For Figure 4f, the interface thickness was set to 3x the diameter and the ionic conductivity of the interface was assumed to be 7.5 times higher than that of the polymeric phase [28]. Interestingly, the total cost for 0.5 µm diameter circles is almost identical to the cost for 1 µm diameter circles. The increase in tortuosity (c.f. Figure 4d) is equalized by the higher SVR. Regarding the dendrite deflection, higher tortuosity would be a favorable choice. In contrast, for rectangles, the bigger structures (1 µm diameter) leading to lower cost (higher ionic conductivity). Since the SVR is lower for larger structures (0.5 µm diameter), the decisive parameter appears to be the lower tortuosity. This confirms the tortuosity trend of the three‐dimensional structure in Figure 4e. For both morphologies, a minimum in cost at 10 vol.% and 20 vol.% for circles and rectangles, respectively, is visible. Literature values for such trends can be found, but also with minima at lower volume fractions. While for Al‐LLZO particles, the highest conductivity is reached at around 40 wt.% (i.e. ∼14 vol.%) for particles in the nm‐range [28], for micro sized Ta‐LLZO particles 10 wt.% (i.e. ∼3 vol.%) was reported [26, 27] (assuming a density of 5 g cm^−3^ for LLZO‐based fillers). This could either be based on a larger interface for Ta‐LLZO particles, shifting the maximum ionic conductivity to lower values or larger difference between the ionic conductivity of the interface compared to the polymeric phase.

## 4 Electrochemical Characterization

### 4.1 Symmetric Cell Characterization

**FIGURE 5:**
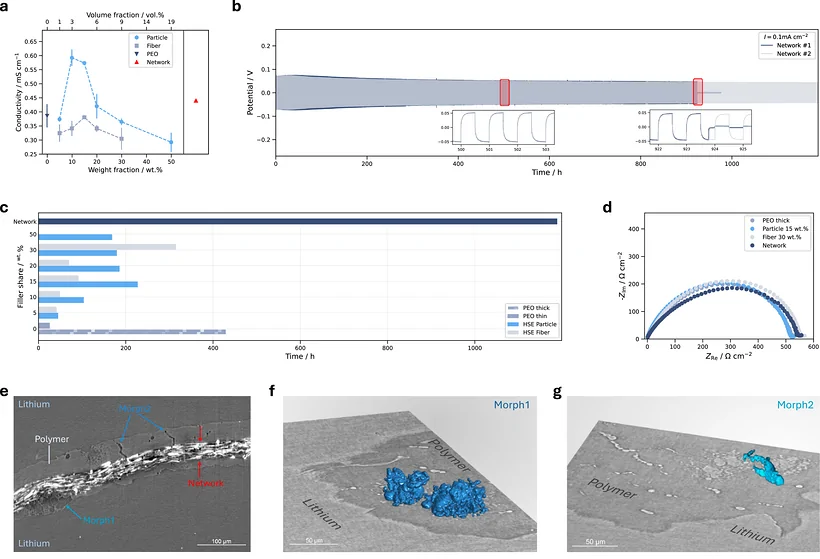
Electrochemical characterization of various HSEs with different filler in symmetric lithium cells. (a) Total ionic conductivities for particles and fibers with different weight fractions and for a network HSE at 60°C. (b) Symmetric cycling of two different symmetric cells with a network HSE at 60°C with 0.1 mA cm^−2^. (c) Comparison between the maximum symmetric cycling duration without a hard short circuit at 60°C with 0.1 mA cm^−2^. (d) Nyquist plot of the EIS measurement for different HSEs. All curves were shifted to *Z*_Re_ = 0 Ω cm^−2^ for *Z*_Im_ = 0 Ω cm^−2^ for better visibility of the solid lithium/solid electrolyte/lithium interface resistance. (e) Cross section of the post‐mortem state of the network #1 from Figure 5b recorded with µXCT. (f,g) Segmented and rendered, three‐dimensional dendritic structure of the post‐mortem state of network #1 from Figure 5b captured with µXCT. While the structure in Figure 5f is connected to a lithium electrode, the structure in Figure5g is not.

The bulk ionic conductivities (Figure 5a) of the different HSEs with various fillers are determined by electrochemical impedance spectroscopy (EIS) in combination with a thickness measurement of each solid electrolyte (Figure S5). Although the network HSE accounts for a high volume fraction, a decent ionic conductivity in the range of 0.44 mS cm^−1^ is reached. This can be explained by the porous structure that enables pathways through the fibers, lowering the tortuosity while creating a high interfacial area. A fiber weight fraction of 15 wt.% (i.e. ∼4 vol.%, for conversion between vol.% and wt.% see Table S1) shows the highest ionic conductivity and is in the same range (0.35–0.4 mS cm^−1^) as PEO without filler. With particles as fillers, the ionic conductivity reaches its maximum at 15 wt.% at 0.64 mS cm^−1^. For both fillers, the simulated trend of first increasing and a subsequent decreasing ionic conductivity with higher filler fraction is confirmed and follows the theory of decreased ionic conductivity due to higher tortuosity. Comparing the thicknesses (Figure S5) of the different HSEs reveals thicker electrolytes with particle fillers compared to HSEs with the same filler fraction of fiber for all filler fractions. Despite the general larger thickness, the total resistivities of particle HSEs are in the same magnitude as those of fiber HSE for low filler fraction (5–15 wt.% (∼1–4 vol.%)). At higher filler fractions (20 and 30 wt.% (i.e. ∼6 and 9 vol.%)), the total resistivity is even lower for fibers compared to particles, which could be caused by particles staying at the surface while fibers are able to be covered completely by polymer. Since all measurements were conducted at temperature (60°C) close to the melting temperature of the used PEO, the solid polymer is already entirely in the amorphous phase and additional filler cannot impede the less conductive (compared to amorphous phase) crystalline phase. However, the ionic conductivity can be improved due to better salt dissociation or by improving the ion mobility. Better salt dissociation leads to more charge carriers, which is directly proportional to the ionic conductivity. To further investigate the effect of the filler on the ionic hopping, the activation energies for all synthesized solid electrolytes were determined by temperature dependent EIS measurements (Figure S8). Since the activation energy at 60°C is in the same order for all electrolytes (0.4–0.45 eV, Table S2), including pure PEO, the energy necessary for ionic hopping is not significantly reduced. This implies that an improved ionic conductivity may be achieved through better salt dissociation leading to an increase in charge carriers or creating additional transport pathways. The ionic conductivity of the filler‐based HSE is on average lower than that of pure PEO, indicating filler clustering which leads to a low SVR but still (compared to pure PEO) increased ion tortuosity, ultimately lowering the ionic conductivity. This theory is supported by the improved ionic conductivity of the network HSE despite higher filler fraction: with one‐piece network filler, clustering is not possible. In addition, it should be noted that some of the error bars are relatively large compared to the differences in ionic conductivities, which limits the interpretive value of the overall comparison. All tested HSE systems (Figure S9 and Table S2), including the network, 15 wt.% particle‐ and 10 wt.% fiber‐based HSE, show a larger lithium transference number $\left(t_{Li^{+}}\geq 0.31\right)$ than pure PEO with LiTFSI $\left(\;t_{Li^{+}}=0.2\right)$, leading to reduced concentration polarization enabling lower overpotential and improved uniform lithium deposition [54].

Figure 5b demonstrates the cycling performance of the developed network HSEs in symmetric lithium metal cells at 60°C with a current density of 0.1 mA cm^−2^. The network HSE performs stable cycling for over 1100 h with a low voltage polarization. Its initial potential constantly decreases and stabilized to 50 mV indicating enhanced interfacial contact with lithium, which is verified with in situ EIS measurements during symmetric cycling (Figure S10). While the HSE/lithium metal resistance decreases within the first 24 h, it increases again after 36 h due to the reaction between PEO and lithium metal [55]. Based on the ionic conductivity (0.44 mS cm^−1^), the total resistance for two lithium/HSE interfaces (420 Ω) and a thickness of 130 µm HSE, the expected potential for applied 0.1 mA cm^−2^ is 36 mV. This value matches the magnitude of the experimental value and demonstrates no soft short circuit was formed during the cycling. For comparison with other fillers, symmetric cells with different filler fractions of fibers and particles were assembled and tested under the same conditions. Figure 5c shows the total stable cycling duration without hard and soft dendrites (based on the cycling curves, Figure S6) of the best‐performing cells (of a total of three assembled cells of each morphology and fraction). Compared to pure thin PEO, both filler morphologies enhance the cycling life with all filler fractions. Interestingly, for particle‐based HSEs, the best‐performing one was the one with the highest ionic conductivity (15 wt.%), while the best one with fibers was the one with the highest filler fraction (30 wt.%). While a high filler fraction improves mechanical strength and dendrite resistance, higher ionic conductivity lowers the polarization. A general connection between thicker HSE and extended cycle life cannot be made for HSEs. With 50 wt.% particles, the cycling performance is limited despite having a high thickness, which is an indication for a rough surface, that possibly causes point contacts when rising the filler fraction. Moreover, increasing the filler fraction is limited by the sphere packing, which theoretically can reach a maximum 74 vol.% but with randomly packed spheres only (63.5 vol.%) which correlates to ∼92 and ∼88 wt.% Ta‐LLZO filler, respectively, in a PEO+LiTFSI system [56]. This also implies that a sufficient amount of organic electrolyte is necessary to fill all interstitial spaces, otherwise, vacant spaces occur, lowering the ionic conductivity and creating low energy dendrite penetration pathways. Without fillers, for pure PEO solid electrolytes with a smooth surface, thicker membranes increase the cycle lifetime drastically from <100 h (50 µm) to >400 h (107 µm). This emphasizes the importance of thickness and its consideration, especially in symmetric cells, where thicker membranes can buffer dendrite growth, thereby impeding short‐circuiting despite uneven lithium plating.

The impedance spectra (Figure 5d) from the best‐performing cells reveal an interfacial resistance for both lithium/solid electrolyte interfaces of 500–570 Ω cm^−2^ for all prepared hybrid solid electrolytes at 60°C. The PEO layers on top of and underneath the network enabling a low interfacial resistance, despite the rigid core network structure. A slight increase in interface resistance with higher filler fractions (e.g. 30 wt.% fiber) is recognizable.

Post‐mortem µXCT investigations (Figure 5e) of symmetric cells were performed to understand the mechanisms that cause the worse‐performing cells, like network #1 from Figure 5b, but also that cause the superior performance of the network structure in general. The solid electrolyte thickness is less than 100 µm, indicating an electrolyte compression after assembly or during cycling. The latter assumption is supported by the shift of bulk resistance to lower values over time during the in situ EIS measurement (Figure S10b), resulting in decreased polarization. Besides this, two different degradation morphologies are visible, labeled as Morph1 and Morph2. Morph1 can be assigned to the typical dendritic structures as reported by Harry et al. [57] for solid polymer electrolytes. Three‐dimensional segmentations of such structures reveal their bubble‐like morphology (Figure 5f), but more importantly, it shows a connection to the lithium metal electrode. Such a connection to the lithium metal electrode is given for every Morph1 structure. Thus, it can be concluded that those structures are filled with lithium, which is supported by the intensity value being the same as for lithium on top of and underneath the electrolyte. On the contrary, the second occurring morphology, Morph2, exhibits an elongated three‐dimensional structure (Figure 5g) and has no visible connection to the lithium metal electrode. Furthermore, those structures are characterized by a darker intensity value, which indicates less attenuating material. This could be attributed to gas formation, like H_2_ evolution during cycling at higher voltages [58]. In general, Morph1 and Morph2 are blocked by the ceramic network. Every interconnected degradation structure remains always on one side of the electrolyte and does not penetrate the network structure, thus does not occur in the opposite polymer layer. Overall, decomposition structures like Morph1 and Morph2 could be caused by residual solvents which can leverage decomposition phenomena in contact with lithium [55]. Comparing network #1 and network #2 from Figure 5b reveals significantly fewer Morph1 and Morph2 structures inside network #2. Fewer degradation structures decrease the probability of a lithium filled one, that penetrates through the electrolyte. One possible explanation for smaller number of degraded areas is fewer reaction or decomposition products within the electrolyte or at the lithium interface due to different batches. While the ceramic network can effectively block dendritic structures, single fillers could also act as obstacles for lithium growth [36]. However, it was found that individual ceramic particles inside a polymer matrix may move due to mechanical stress, lowering the blocking capability [59].

### 4.2 Full Cell Characterization

**FIGURE 6:**
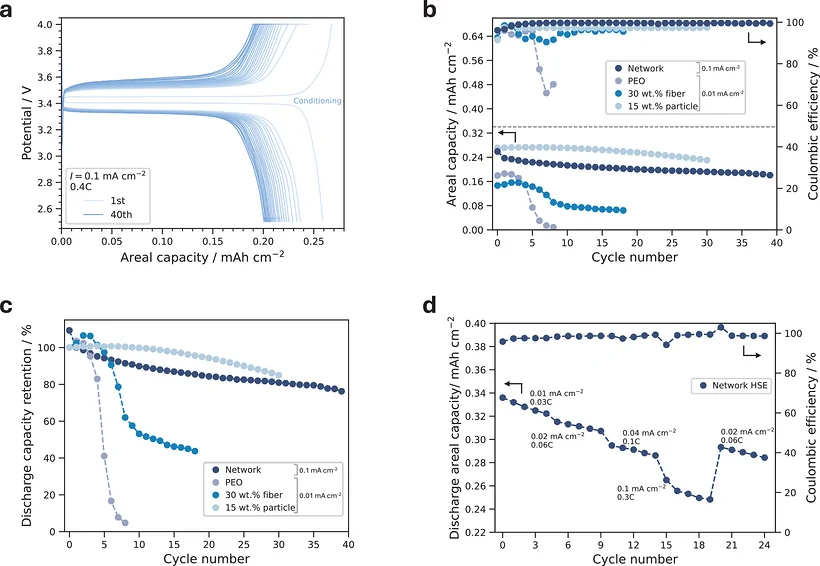
Electrochemical cycling in LFP/solid electrolyte/Li cell configuration. (a) Charge−discharge potential profiles of a network HSE. A conditioning cycle is performed with 0.01 mA cm^−2^ before charging and discharging with 0.1 mA cm^−2^. (b) Cycling performance of different HSEs as well as pure PEO solid electrolyte. While the network is cycled at 0.1 mA cm^−2^, all other electrolytes are cycled at 0.01 mA cm^−2^. (c) The according discharge capacity retentions from cells cycled in Figure6b. For the network HSE the first cycle counts as conditioning and is thus above 100 %. (d) Rate capability test of a ceramic network HSE cell. All tests were performed at 60°C.

Cycling of symmetric lithium metal cells allows only limited evaluation of the electrolyte performance. An applied current density of 0.1 mA cm^−2^ for 30 min leads to 0.24 µm (assuming homogeneous plating) plated/stripped lithium and 0.05 mAh cm^−2^ of capacity, respectively. In contrast, a reasonable battery capacity significantly exceeds 0.05 mAh cm^−2^. Therefore, the network HSE is also evaluated in a full cell configuration with a lithium anode and composite LFP cathode. The cells are charged until 4 V to limit potential decomposition of PEO at higher voltages [60]. For comparison, full cells with the best performing fiber (30 wt.%) and particle (15 wt.%) fraction, as well as the solid polymer alone were tested. After a condition cycle (0.01 mA cm^−2^), the Li/network HSE/LFP cell was able to cycle stably for 40 cycles with a current density of 0.1 mA cm^−2^ at an initial areal capacity of 0.25 mAh cm^−2^, representing a C‐rate of 0.4 C. No voltage noise due to soft dendrites, which is typical of PEO‐based solid electrolytes [61], was visible (Figure 6a). This indicates homogeneous lithium deposition and dissolution at the anode side, which suggests the effectiveness of the dendrite blocking network at reasonable capacities. A constantly high coulombic efficiency of > 99 % after the third cycle indicates a high charge‐discharge reversibility (Figure 6b). In contrast, all other filler morphologies, as well as the pure thick PEO solid electrolyte could not be cycled with comparable capacities at the same current density. For the 15 wt.% particle‐based HSE, the full cell shows stable cycling at 0.01 mA cm^−2^ but with fast capacity fading, as can be seen in Figure 6c. In addition, the coulombic efficiency was only 97.5% at maximum. Likewise, the capacity of the network full cell decreased quickly, leading to a capacity retention of 76.2% of its first cycle discharge capacity after 40 cycles. However, this behavior is typically seen for PEO systems, including a PEO‐based LFP cathode [62]. The 30 wt.% fiber‐based HSE and pure, thick PEO solid electrolyte performing even worse at low capacity (> 0.2 mAh cm^−2^) and low current densities (0.01 mA cm^−2^), losing more than 50% of their initial capacities before reaching the tenth cycle due to severe dendrite formation. The potential vs. capacity curves (Figure S11) show severe soft short circuit issues caused by dendrite formation for the pure PEO and 30 wt.% fiber‐based HSE but also for the 15 wt.% particle‐based HSE, as indicated by the fluctuation of the charging voltages. Rate capability tests (Figure 6d) of the network full cell configuration show the common faster capacity loss at higher charging/discharging rates. Higher capacities can again be achieved after switching back to lower current densities, with decent coulombic efficiencies.

A general weak point of single‐filler HSE is a possible rough surface due to fillers located at the surface, which could cause increased interfacial resistance. If the fillers are not contributing to the ionic transport, due to a high interfacial resistance between the polymer and inorganic phases, fillers at the surface lower the active area for ionic transport and thus also increasing the local current density. This event is avoided with a single, connected filler like the ceramic network which incorporates pure PEO layers underneath and above the network. The ceramic network could be even more beneficial if a polymer matrix or surface modification enables ion hopping between the ceramic and polymer phases or increases the higher conductive interfacial area. Especially enabling the cross‐phase ion hopping could leverage the performance of LLZO‐based HSE drastically. However, the simulation and experimental conductivity results confirm the assumption that no long‐range ionic diffusion between the inorganic and organic phases is taking place. An alignment of the network perpendicular to the electrodes, instead of the designed parallel orientation, could benefit the ionic conductivity but worsen the dendrite resistance due to lower tortuosity. In addition, the thickness of both polymer layers could be further decreased, lowering the voltage polarization and volume that can undergo reaction leading to dendritic structures, that are prone to be penetrated by lithium metal. Besides all this, it needs to be noted that all measurements were performed at elevated temperature (60°C), thus reducing improvement due to the filler effect compared to a pure polymer solid electrolyte, which would be more pronounced at room temperature.

## 5 Conclusion

A Ta‐LLZO‐based aligned, porous, ceramic network hybrid solid electrolyte was developed. Its microstructural characterization revealed an aligned ceramic network with a high SVR (1.02 m^2^ g^−1^). EIS measurements and symmetric cycling tests support the simulation‐based findings: Particle fillers lead to the highest ionic conductivity (0.64 mS cm^−1^, 60 °C, 15 wt.%) and the network structure can effectively block dendritic structures due to high tortuosity, leading to superior symmetric cycling performance of > 1100 h with 0.1 mA cm^−2^ at 60°C and low polarization. Similarly, the network structure enables improved cycling performance in full cells with LFP cathodes compared to other Ta‐LLZO‐based filler morphologies and fractions. Structures with bubble‐like morphology connected to the lithium electrode and elongated structures with no connection to the lithium electrode were detected by post‐mortem µXCT scans, suggesting initial gas formation due to polymer decomposition that creates lithium penetration pathways. In summary, this study emphasizes the importance of tortuosity consideration for both ionic conductivity and dendrite suppression, and discusses different filler morphologies, fractions and sizes to serve as a guideline for advancing hybrid solid electrolytes for lithium metal batteries.

## 6 Experimental

### 6.1 Synthesis of Ta‐LLZO Filler

A Li_6.6_La_3_Zr_1.6_Ta_0.4_O_12_ precursor solution was synthesized via a sol–gel process. Stoichiometric amounts of metal nitrates as the reagents (LiNO_3_ (Alfa Aesar, USA), La(NO_3_)3∙6H_2_O (Sigma–Aldrich, USA), Zr(OCH_2_CH_2_CH_3_)_4_ (Sigma–Aldrich, USA), C_10_H_25_O_5_Ta) and ethanol absolute (Merck, Germany) and 2‐methoxy (Th. Geyer, Germany) as the solvent were mixed and stirred under applied heat for several days until a clear, brownish solution was obtained. To compensate for the lithium loss during calcination, an extra amount of 50 wt.% LiNO_3_ was added. As a polymeric carrier solution to make electrospinning with the precursor solution possible, 11.5 wt.% polyvinylpyrrolidone (PVP) (Thermo Scientific, USA, M_w_ = 1 300 000) was mixed with dimethylformamide (DMF) (VWR, USA) and stirred for 1 day. The polymer solution was mixed in a 6:5 (PVP/DMF:Ta‐LLZO precursor) volume ratio and stirred for 5 h. Electrospinning (IME Medical Electrospinning, Netherlands) was carried out in a controlled environment at 25°C with 45 % relative humidity. The electrospinning solution was injected via a needle at a feeding rate of 12 µl min^−1^ and 180 mm away from the rotating drum collector operating at 200 rpm. While the electrical potential at the needle is −4 kV, it is set to 22 kV at the drum collector. To obtain a sufficient thick fiber mat, 6 mL of solution were spun.

After electrospinning, single pieces of the fiber mats were placed between two MgO plates with two spacer plates in between to avoid bending of the fiber mat without applying pressure on the mat. The loaded MgO plates were placed inside an Al_2_O_3_ crucible (Al‐contamination of LLZO structure) and covered with a lid for calcination, which was conducted inside a muffle furnace (Nabertherm, Germany) with the following heat protocol. First, the sample was heated from room temperature until 200°C (5°C min^−1^, 1 h rest). Afterward, the heat ramp was lowered to 1°C min^−1^ until 350°C and increased again to 5°C min^−1^ until 750°C, which was held for 3 h. The furnace cooled down naturally. After calcination the fibers were directly transferred into an argon filled glovebox (MBraun, Germany (O_2_ <0.1 ppm, H_2_O <0.1 ppm)).

Al‐contaminated and Ta‐substituted LLZO particle filler (Figure S7a) were synthesized according to the synthesis route reported in previous publications [51, 63]. The obtained pellets were crushed into µm‐sized powder using a motor grinder RM 200 (Retsch, Germany) with a tungsten carbide crucible and pestle for 1 h. Single fiber fillers (Figure S7b) were produced by manually crushing the network with a mortar.

### 6.2 Preparation of Hybrid Solid Electrolyte

Polyethylene oxide (PEO) (Sigma–Aldrich, USA; 4 × 10^6^ g mol^−1^, at 50°C for 2 d under high vacuum (Büchi, Switzerland)) with lithium bis(trifluoromethanesulfonyl)imide (LiTFSI) (IoLiTec, Germany; dried at 120°C for 2 d under high vacuum) were used as the polymer backbone for the hybrid solid electrolytes. PEO and LiTFSI were mixed at an EO:Li ratio of 14:1 and dissolved in acetonitrile (VWR, USA) under continuous magnetic stirring for 1 day. The polymer solution was either cast onto the fiber network or mixed with single fibers or particles. To obtain homogeneously distributed fibers and particles in the polymer solution, a planetary mixer (Thinky, USA) was used. Both castings (onto the fiber network and of the polymer‐fiber solution) were done inside a polytetrafluoroethylene (PTFE) dish. For vacuum infiltration of the fiber network, the PTFE dish was placed inside a desiccator and put under vacuum after casting. To evaporate the residual solvent, the hybrid solid electrolytes were dried under argon atmosphere for several days first under atmospheric pressure and then with lowered pressure (< 1 kPa). The resulting hybrid solid electrolyte films were stored and processed inside the glovebox.

### 6.3 Preparation of Composite Cathodes

To test the solid electrolyte performance in full cells, LiFePO_4_‐based (LFP) composite cathodes were synthesized. First, LFP powder (MSE Supplies, USA (vacuum dried at 50°C for 4 d)) was mixed and ground with 10 vol.% carbon black (Alfa Aesar, USA (vacuum dried at 50°C for 4 d)). The mixture was further mixed with PEO and LiTFSI (EO:Li ratio 14:1) in a (50:50)(PEO:LFP+CB) vol.% ratio. After dry mixing, 5 mL acetonitrile per 0.5 g LFP was added and mixed under continuous stirring for 1 day. The resulting slurry was mixed for 5 min at 1500 rpm in the planetary mixer. After mixing, the slurry was either tape‐cast with a doctor blade on aluminum foil or drop‐cast on a copper current collector and dried at 80°C under an argon atmosphere for at least 1 d.

### 6.4 Microscopical and Structural Characterization

For the SEM analysis, a FEI Quanta FEG 650 (FEI, USA) was used. The samples were coated with a < 100 nm thick Au layer via sputter coating (Cressington, UK) to avoid charge accumulation artifacts from the electron beam. The Nano XCT scan was performed with an Xradia Ultra 810 (ZEISS, Germany) in air at high resolution with 65 nm voxel size. A maximum energy of 5.4 keV and 700 projections with an exposure time of 90 s were used. The µXCT scans were recorded with an Xradia 620 Versa (ZEISS, Germany). An acceleration voltage of 40 kV and 1601 projections were used. To obtain sufficient photon flux, the exposure time was set to 15 s. High‐magnification measurement was performed with a 20x objective resulting in a voxel size of 0.70 µm. Prior to µXCT post‐mortem measurements the samples were removed from the cell housing and encapsulated with solvent‐free resin‐based glue to ensure airtightness and avoid photon attenuation from the cell housing. Energy‐dispersive x‐ray spectroscopy (EDS) and Raman spectroscopy measurements were carried out in combination with a Gemini 560 (ZEISS, Germany). For EDS, an Ultim Max detector (100 mm^2^, Oxford Instruments, UK) and an accelerating voltage of 10 kV at a working distance of 8.5 mm were used. Aztec software was utilized for EDS data analysis. Raman spectroscopy was performed with a WiTec Raman microscope, using solid‐state lasers with an excitation wavelength of 532 nm with 600 grating per mm. XRD measurements were performed using an Empyrean DY 2785 x‐ray diffractometer (Malvern Panalytical, UK) with a Cu x‐ray source (1.54 Å) in reflection mode from 5° to 90° 2θ. The surface area was measured with a gas adsorption analyzer (Quantachrome Instruments, USA) with krypton (4.8, Air Liquide, Germany) at 77.35 K. A degassing time of 4 h at 200°C and a saturation vapor pressure of 0.35 kPa were used. The BET range was set to 0.1> P/P0> 0.3 and the cross‐sectional area to 20.5 Å^2^. MS coupled TGA was conducted on an TGA/STA‐QMS 403D thermoanalyzer (Netzsch, Germany) between 25°C and 1000°C with a heating rate of 5°C min^−1^ under N_2_/O_2_ (80/20) atmosphere. Three‐dimensional renderings of µXCT scans were created with Dragonfly software.

### 6.5 Electrochemical Characterization

For electrochemical characterization, the symmetric and full cells were assembled in stainless steel *Swagelok*‐type cells with an inner diameter of 13 mm (without insulating polyethylene terephthalate foil). Copper plates (Ø 12 mm) were used as current collectors. Lithium metal as electrode had a diameter of 10 mm, while the HSEs were punched to a diameter of 12 mm for symmetric and 13 mm for full cells with a 12 mm LFP@Aluminum cathode. All electrochemical tests, including symmetrical cycling, electrochemical impedance spectroscopy (EIS) and full cell cycling were carried out using a VMP3 potentiostat (BioLogic, France) at 60°C inside a climate chamber (Binder GmbH, Germany). Before symmetric cycling and EIS measurements, the cells were equilibrated for 2 h and for 24 h before full cell cycling inside the climate chamber at 60°C. EIS data were collected within a frequency range of 1 Hz–1 MHz with a 12 mV amplitude.

### 6.6 Simulation

The simulations were performed based on customized python scripts working with the NumPy library. For tortuosity determination, the library TauFacator with periodic boundary conditions was utilized [64]. The 3D tortuosity simulations were based on a cubic array with 600^3^ entries.  pixel/voxel size (one array entry) was assigned to 25 nm for better readability. The long cylinders of the aligned network structure were randomly tilted between 0° and 20° and the simulation direction was set perpendicular to the fiber alignment. For 2D conductivity simulations a 1000^2^ array with periodic boundary conditions was used. The fillers were distributed randomly and no overlap between different fillers was assumed. The ionic hopping with a certain activation energy was simulated as hopping from one entry to another entry, allowing vertical and horizontal jumps in one direction, while each jump to another array entry added to the total cost (activation energy, Figure 4g). Polymer‐labeled array entries and interface‐labeled entries were assigned different costs. No hopping into the ceramic labeled entries was allowed. An implementation of Dijkstra's algorithm was used to find the cheapest/lowest activation energy (weighted graph) pathway from one side to the other. Each point on one side was simulated and the mean value was taken as result.

## Author Contributions

**Luca Weckelmann**: Writing – original draft, Writing – review & editing, Visualization, Validation, Software, Methodology, Investigation, Formal analysis, Data curation, Conceptualization. **Jeong Seop Yoon**: Writing – review & editing, Validation. **Jehad Ahmed**: Formal analysis, Investigation, Writing – review & editing, Validation. **Krzysztof Dzieciol**: Supervision, Writing – review & editing, Validation. **Anna Windmüller**: Writing – review & editing, Validation. **Luc Raijmakers**: Writing – review & editing, Validation. **Sanja Tepavcevic**: Writing – review & editing, Validation, Project administration. **Hans Kungl**: Writing – review & editing, Validation. **Venkat Srinivasan**: Writing – review & editing, Validation, Funding acquisition, Project administration. **Chih‐Long Tsai**: Supervision, Conceptualization, Writing – review & editing, Validation, Project administration. **Rüdiger‐A. Eichel**: Supervision, Funding acquisition, Project administration, Resources, Writing – review & editing.

## Funding

This work was supported by the Federal Ministry of Research, Technology and Space (BMFTR) and the US Department of Energy (DOE) from the project “US‐German Cooperation on Energy Storage” under the funding program “LiSI‐2‐Lithium‐Solid‐Electrolyte Interfaces” [grant number 13XP0509A] and by “Ministerium für Kultur und Wissenschaft des Landes Nordrhein‐Westfalen” from the project “High Performance Solid‐State Batteries” (HIPSTER).

## Conflicts of Interest

The authors declare no conflicts of interest.

## Supporting information




**Supporting File**: advs76063‐sup‐0001‐SuppMat.docx.

## Data Availability

The data that support the findings of this study are available from the corresponding author upon reasonable request.
