# Supplementary material for: A Ceramic Network for Hybrid Solid Electrolyte Lithium Metal Batteries
Source: Adv Sci (Weinh). 2026 Jul 2;13(50):e76063. doi: 10.1002/advs.76063 (PMC13337035; doi:10.1002/advs.76063)
Supplement: Supplementary file 1 [file ADVS-13-e76063-s001.docx]

**Supporting Information**

*A Ceramic Network for Hybrid Solid Electrolyte Lithium Metal Batteries*

Luca Weckelmann^1,2,*^, Jeong Seop Yoon^3^, Jehad Ahmed^1,2^, Krzysztof Dzieciol^1^, Anna Windmüller^1^, Luc Raijmakers^1^, Sanja Tepavcevic^4^, Hans Kungl^1^, Venkat Srinivasan^5^, Chih-Long Tsai^1,*^, Rüdiger-A. Eichel^1,2^

*^1^ Institute of Energy Technologies, Fundamental Electrochemistry (IET-1), Forschungszentrum Jülich, D-52425 Jülich, Germany
^2^ Institute of Physical Chemistry, RWTH Aachen University, D-52074 Aachen, Germany
^3^ Chemical Sciences and Engineering Division, Argonne National Laboratory, Lemont, IL 60439, USA
^4^ Materials Science Division, Argonne National Laboratory, Lemont, IL 60439, USA
^5^ Argonne Collaborative Center for Energy Storage Science, Argonne National Laboratory, Lemont, IL 60439, USA*

***Li^+^ Transference Number Measurement***

Li^+^ transference numbers were calculated according to the methodology introduced by Bruce et al.[1] based on chronoamperometry[2]. EIS data of symmetric Li/solid electrolyte/Li cells were collected within a frequency range of 1 Hz-1 MHz with a 12 mV amplitude at 60 °C before (after 2 h of equilibration at 60 °C) and after direct current (DC) polarization measurement with ∆*V* = 5 mV for 7 hhvh. The total interfacial resistances (solid electrolyte/lithium interfaces) *R*_0_ before and *R*_SS_ after DC polarization were obtained from fitting the EIS measurements. With the initial current *I*_0_ and the steady state current *I*_SS_ after 7 h the transference number was calculated using Equation (1). The results are listed in Table S2.

$$t_{\mathrm{Li}^{+}}=\frac{I_{\mathrm{SS}}\left( \Delta V-I_{0}R_{0} \right)}{I_{0}\left( \Delta V-I_{\mathrm{SS}}R_{\mathrm{SS}} \right)}$$

(1)

***In situ EIS***

Symmetric cycling with *in situ* EIS measurements were performed in Li/HSE network/Li cells at 60 °C. EIS data were collected within a frequency range of 1 Hz-1 MHz with 12 mV amplitude first in the pristine state before cycling, then every 3 h and finally every 20 h with a 1 h break within the symmetric cycling.

***Activation Energy***

For determination of the activation energy, EIS measurements (VSP-300 potentiostat, BioLogic, France) at different temperatures were conducted. The solid electrolytes were placed between stainless steel current collectors inside *Swagelok*-type cells and equilibrated at elevated temperatures for several hours before measurement. The set temperature was held for at least 1 h before each measurement inside a temperature chamber (Vötsch, Germany). EIS data (Figure S10a) were collected within a frequency range of 1 Hz - 7 MHz with 12 mV amplitude and fitted with an equivalent circuit to obtain the total ionic conductivities. Using the slope of the linear fits in Figure S10b the activation energies were calculated based on the Arrhenius equation. The results are listed in Table S2.

**Figure S1:** Mass spectroscopy measurement that is coupled with the TGA measurement in Figure 1d.

**Figure S2:** Element assignment for the different energy peaks of the EDS measurement in Figure 2c.

**Figure S3:** Two-dimensional cross section of the network HSE recorded with µXCT. The scale bar equals 100 µm.

**Table S1:** Overview of the solid electrolytes synthesized for this work. The ether oxygen to lithium ratio (EO:Li) was set to 14:1 for all solid electrolytes. The weight fraction of the filler is related to the total weight of PEO+LiTFSI+filler. For conversion of weight percentage to volume percentage a filler density of 5.3 g cm^−3^ for Ta-LLZO is assumed.

| Polymer | Salt | Filler material | Filler type | Weight fraction / wt.% | Volume fraction / vol.% |
| --- | --- | --- | --- | --- | --- |
| PEO | LiTFSI | Ta-LLZO | Particle, Fiber | 5 | ~ 1 |
| PEO | LiTFSI | Ta-LLZO | Particle, Fiber | 10 | ~ 3 |
| PEO | LiTFSI | Ta-LLZO | Particle, Fiber | 15 | ~ 4 |
| PEO | LiTFSI | Ta-LLZO | Particle, Fiber | 20 | ~ 6 |
| PEO | LiTFSI | Ta-LLZO | Particle, Fiber | 30 | ~ 9 |
| PEO | LiTFSI | Ta-LLZO | Particle | 50 | ~ 19 |
| PEO | LiTFSI | Ta-LLZO | Network | / | / |
| PEO | LiTFSI | / | / | 0 | 0 |

**Figure S4:** Results from the two-dimensional cost simulation for circle as fillers. a) Minimum shift for different interphase thicknesses. A factor of 2x relates two an interphase thickness of two times the diameter of the filler. b) Minimum shift for different ionic conductivity *σ* relations between the polymer phase and interphase.

**Figure S5:** Solid electrolyte thicknesses measured before cell assembly (top) and bulk resistance (bottom).

**Figure S6:** Symmetric lithium metal cell cycling of HSEs with different filler share (left row: particles, right row: fiber) at 60 °C with 0.1 mA cm^−2^. Herein, the cell with the longest cycle life was chosen out of three assembled cells for each HSE.

**Figure S7:** SEM images of Ta-LLZO filler. a) Ta-LLZO ceramic µm-particles. b) Ta-LLZO ceramic single µm-fibers.

**Figure S8:** Determination of activation energies. a) Exemplary Nyquist plot of a network HSE at different temperature fitted with the equivalent circuit L-R1-R2//CPE1- R3//CPE2-W (L: inductor, R: resistor, CPE: constant phase element, W: Warburg element). b) Total ionic conductivities obtained from the EIS measurements as a function of temperature. The data were plotted such that the slope of a linear fit yields the activation energy according to the Arrhenius equation.

**Table S2:** Activation energies and transference numbers for different hybrid and pure polymer solid electrolytes. The activation energy values are obtained from Figure S8 by linear fitting and divided into two regions before and after melting temperature of PEO (60 °C). The transference numbers are calculated based on the measurements from Figure S9 with Equation (1).

| HSE Type | Weight fraction | Weight fraction | Activation energy / eV | | Transference number |
| --- | --- | --- | --- | --- | --- |
|  | / wt.% | / wt.% | < 60 °C | ≥ 60 °C |  |
| Particle | 5 | ~ 1 | 0.95 | 0.40 | / |
| Particle | 10 | ~ 3 | 0.83 | 0.44 | 0.31 |
| Particle | 15 | ~ 4 | 0.92 | 0.41 | / |
| Particle | 20 | ~ 6 | 0.95 | 0.45 | / |
| Particle | 30 | ~ 9 | 1.00 | 0.44 | / |
| Particle | 50 | ~ 19 | 1.03 | 0.44 | / |
| Fiber | 5 | ~ 1 | 0.91 | 0.41 | / |
| Fiber | 10 | ~ 3 | 0.98 | 0.44 | / |
| Fiber | 15 | ~ 4 | 0.94 | 0.42 | 0.48 |
| Fiber | 20 | ~ 6 | 0.97 | 0.42 | / |
| Fiber | 30 | ~ 9 | 0.94 | 0.43 | / |
| Network | / | / | 1.00 | 0.43 | 0.32 |
| Pure PEO | / | / | 0.89 | 0.42 | 0.20 |

**Figure S9:** Determination of Li^+^ transference numbers at 60 °C. a) DC polarization curve of Li/solid electrolyte/Li cells with 5 mV voltage. b) Nyquist plots of the EIS measurements before and directly after DC polarization experiments from a).

**Figure S10:** Symmetric cell cycling and it’s *in situ* EIS measurements at 60 °C. a) Symmetric cycling of a Li/network HSE/Li cell. The red dots represent the interruptions by EIS measurements. b) Nyquist plot of the EIS measurements from a). The measurements after >0 h were shifted upward along the -*Z*_Im_-axis for better readability.

**Figure S11:** Charge−discharge potential profiles of LFP/solid electrolyte/Li cell configurations. A conditioning cycle is performed with 0.01 mA cm^−2^ before charging and discharging with 0.1 mA cm^−2^ for all cycling tests. a) Thick PEO as solid electrolyte. b) HSE with 15 wt.% LLZTO particles. c) HSE with 30 wt.% LLZTO fiber. All tests were performed at 60 °C.

**References**

[1]	P.G. Bruce, J. Evans, C.A. Vincent, Conductivity and transference number measurements on polymer electrolytes, Solid State Ionics 28–30 (1988) 918–922. https://doi.org/10.1016/0167-2738(88)90304-9.

[2]	D. Xu, J. Su, J. Jin, C. Sun, Y. Ruan, C. Chen, Z. Wen, In Situ Generated Fireproof Gel Polymer Electrolyte with Li6.4Ga0.2La3Zr2O12 As Initiator and Ion-Conductive Filler, Advanced Energy Materials 9 (2019) 1900611. https://doi.org/10.1002/aenm.201900611.
